# Production of Fungal Nanochitosan Using High-Pressure Water Jet System for Biomedical Applications

**DOI:** 10.3390/ma15041375

**Published:** 2022-02-12

**Authors:** Kota Ogura, Clément Brasselet, Gustavo Cabrera-Barjas, Masoud Hamidi, Amin Shavandi, Marguerite Dols-Lafargue, Naoki Sawamura, Cédric Delattre

**Affiliations:** 1 Sugino Machine Limited, 2410 Hongo, Uozu, Toyama 937-8511, Japan; k.ogura@sugino.com (K.O.); n.sawamura@sugino.com (N.S.); 2 Université Clermont Auvergne, Clermont Auvergne INP, CNRS, Institut Pascal, 63000 Clermont-Ferrand, France; clement.brasselet@uca.fr; 3 Unidad de Desarrollo Tecnológico, Parque Industrial Coronel, Universidad de Concepción, Concepción 3349001, Chile; g.cabrera@udt.cl; 4 BioMatter Unit, École Polytechnique de Bruxelles, Université Libre de Bruxelles (ULB), Avenue F.D. Roosevelt, 50-CP 165/61, 1050 Brussels, Belgium; masoud.hamidi@ulb.be (M.H.); amin.shavandi@ulb.be (A.S.); 5 Department of Medical Biotechnology, Faculty of Paramedicine, Guilan University of Medical Sciences, Rasht 44771-66595, Iran; 6 EA 4577 Œnologie, INRA, USC 1366, ISVV, Bordeaux INP, Université de Bordeaux, 33000 Bordeaux, France; marguerite.dols@enscbp.fr; 7 Institut Universitaire de France (IUF), 1 Rue Descartes, 75005 Paris, France

**Keywords:** fungal chitosan, nanofiber, high-pressure water jet system, antimicrobial film, antioxidant film

## Abstract

In this present work, fungal nanochitosans, with very interesting particle size distribution of 22 µm, were efficiently generated in high-yield production using a high-pressure water jet system (Star Burst System, Sugino, Japan) after 10 passes of mechanical treatment under high pressure. The specific characterization of fungal chitosan nanofibers suspensions in water revealed a high viscosity of 1450 mPa.s and an estimated transparency of 43.5% after 10 passes of fibrillation mechanical treatment. The mechanical characterization of fungal nanochitosan (NC) film are very interesting for medical applications with a Young’s modulus (E), a tensile strength (TS), and elongation at break (e%) estimated at 2950 MPa, 50.5 MPa, and 5.5%, respectively. Furthermore, we exhibited that the fungal nanochitosan (NC) film presented very good long-term antioxidant effect (reached 82.4% after 96 h of contact with DPPH radical solution) and very interesting antimicrobial activity when the nanochitosan (NC) fibers are mainly activated as NC-NH_3_^+^ form at the surface of the film with 45% reduction and 75% reduction observed for *S. aureus* (Gram-positive) and *E. coli* (Gram-negative), respectively, after 6 h of treatment. These promising antimicrobial and antioxidant activities indicated the high potential of valorization toward biomedical applications.

## 1. Introduction

Chitosan (CS) is a natural cationic polysaccharide composed of glucosamine and *N*-acetyl glucosamine linked by *β*-(1-4) glycosidic bonds. Chitosan is derived from chitin which is one of the most abundant biopolymers on Earth right after cellulose. The story of chitin began with the discovery of a French scientist in 1811, but later described as a natural poly-*β*-(1-4)-*N*-acetyl-D-glucosamine [1,2]. Chitin crystallize in two different polymorphic forms namely α and β-chitin [2] which have a different crystallographic cell, a γ polymorphic form is also often described as a combination of α and β chitin forms. The chemical structure of native chitin leads to have an insoluble behavior of chitin in water or in other common organic solvents. To overcome this drawback, chitin can be efficiently transformed into chitosan by a hot alkaline treatment [1,2]. The partial deacetylation of initial chitin provides chitosan. The chitin/chitosan frontier is usually fixed at 50% of residual acetyl groups. CS coming from this deacetylation step is soluble in water acidic media (2 < pH < 6). Chitosan has a pKa of 6.2 [1,3]. This behavior allows chitosan to have more industrial applications than chitin. Most of CS world production comes from marine resources (shrimp cells, squid pens, lobsters, and other crustaceans). However, more promising chitosan sources begin to appear on the market, such as fungi or mushroom or insect chitosan [1,2,4]. Fungi and mushroom chitosan are for instance the only category of chitosan that can be used in the treatment of wines [5,6] and the industrial potential of valorization is in continue development [1,2]. Chitosan is a non-toxic and biocompatible polysaccharide [7] with strong biological properties such as antimicrobial or antifungal, wound-healing, cellular-binding for medicine, cosmetic, pharmaceutical, agricultural and food applications [2,8,9,10]. Mostly, the film casting method was largely developed to generate antimicrobial chitosan films from chitosan solution using acid such as lactic acid, acetic acid or HCl [1,2,3,4,5,6,7,8,9,10,11,12,13]. However, in some studies, the antimicrobial effects are principally described by low residual traces of acetic acid in the films [2,7,8,9,10].

Over the last few years, lot of applications were performed using chitin/chitosan nanofibers for biomedical application as novel green nanomaterials [11,12,13]. Generally, polysaccharides nanofibers are mostly well-described as fibers with high aspect ratio and less than 100 nanometers of diameters [11,13,14,15]. As described by Ifuku (2014), chitin and chitosan nanofibers possessed very interesting mechanical, optical, and morphological properties by comparison with the micro-sized fibers [13]. Consequently, a main focus is continuously performed for the industrial scale-up preparation of chitosan/chitin nanofibers and derivatives. Several physicochemical methodologies such as: electrospinning, ultrasonic, grinder technology, and atomizing system using effective high-pressure waterjet systems have been proposed to generate nanochitosan/nanochitin from crustaceous and cell walls mushrooms [16,17,18,19,20]. As for example, in the electrospinning setup, artificial chitosan nanofibers with controlled sizes could be generated using specific interaction between electrically charged chitosan (R-NH_3_^+^) solutions (at pH < 6 in acetic acid and/or lactic acid) and electrically charged metal surface [14,16]. On the other hand, high-pressure waterjet systems were efficiently used to mechanically disintegrate chitosans into thinner nanochitosan fibers using a ball-collision chamber [13,19,20,21].

Even if recently, it was revealed the production of chitin nanofibers from the cell walls of mushrooms using grinder treatment [13,21], as far as we know, few studies were performed on nano-fibrillation of fungal chitosans. Consequently, in this present work, we used the high-pressure water jet system technology (Star Burst System) for the first time on a high molecular weight fungal chitosan from *Agaricus bisporus* biomasses to generate very high-yield nanochitosan fibers with this eco-friendly environmental green process. These new fungal nanochitosan fibers were efficiently used for the synthesis of antimicrobial and antioxidant films with high potential for valorization in biomedical field.

## 2. Materials and Methods

### 2.1. Materials

Fungal chitosan was sourced by BioLaffort (Floirac, France). As previously studied, this chitosan from *Agaricus bisporus* displayed an average molecular weight (Mw) of 400 kDaltons and a degree of acetylation (Dac) estimated by ^1^H-NMR at 15.8% [6].

### 2.2. Preparation of Fungal Chitosan Nanofibers

The dry powder of fungal chitosan was suspended in tap water using homogenizer Ultra Turrax (during 5 min) and 2 wt% slurry was prepared. The fungal chitosan suspension was then processed by Star Burst Mini system (HJP-25001CE, Sugino Machine Co., Ltd., Suzuka, Japan) equipped with a ball-collision chamber, at 2000 bar with 0.12 mm nozzle. In total, 2 passes, 5 passes, 10 passes, and 20 passes processed nanochitosans samples were collected for analysis.

### 2.3. Viscosity Analysis of Fungal Chitosan Nanofibers Suspensions

The viscosity (mPa.s) of the fungal chitosan nanofibers suspension (1 wt% in water) from 2 to 20 passes of treatments with a high-pressure water jet system was analyzed with an AR 2000 rheometer system (TA-Instrument, AR-2000) equipped with a Peltier temperature control system and a cone-plane module [6].

### 2.4. Particle Size Distribution Analysis of Fungal Chitosan Nanofibers Suspension

The particle size distribution of the fungal chitosan nanofibers suspension was analyzed using a Laser Scattering Particle Size Distribution Analyzer Partica LA-960 (Horiba, Ltd., Kyoto, Japan) with a refractive index of 1.510 based on previous work [20].

### 2.5. FE-SEM Analysis of Fungal Chitosan Nanofibers

For field emission scanning electron microscopic (FE-SEM) analysis of fungal chitosan nanofibers, the chitosan nanofibers suspension (10 passes) were washed with t-butyl alcohol (5 times) through a centrifugation (15 min, 10,000× *g*) to remove water. The final pellet was freeze dried to obtain fungal nanochitosans cast film before microscopic analysis. The chitosan nanofiber film was first coated with platinum and finally analyzed using JSM-6700F (JEOL, Ltd., Akishima, Japan).

### 2.6. Light Transmittance Analysis of Fungal Chitosan Nanofibers Suspensions

The light transmittance of the fungal chitosan nanofiber suspensions (nanofibers content of 0.1 wt% in water) was measured at 600 nm using a UV–vis spectrophotometer (Jasco V-730; Paris, France).

### 2.7. Fungal Nanochitosan Film Preparation

For the preparation of nanochitosan (NC) film, the chitosan nanofibers suspension (10 passes) were dispersed in distilled water at a nanofibers content of 0.5 wt%. The diluted suspensions were then vacuum-filtered using a 0.1 µm hydrophobic Polyvinylidene Fluoride (PVDF) membrane (47 mm). The obtained fungal nanochitosan film (sandwich of nanochitosan by two PVDF membrane) was dried under pressure at 50 °C overnight to obtain targeted film thickness of around 50 µm.

### 2.8. Mechanical Properties of Fungal Nanochitosan Film

The determination of the mechanical properties such as: tensile strength TS, Young’s modulus E, and elongation at break (e%) of the fungal nanochitosan film were carried out at 25 °C by using an electromechanical Shimadzu AGS-X universal testing machine. Film of length/width = 30 mm/5 mm have been mechanically tested in triplicate at a speed of 1 mm/min with a 5 kN load cell system.

### 2.9. In Vitro Biological Activity of Fungal Nanochitosan Film

#### 2.9.1. Antioxidant Activity

The antioxidant activity of the fungal nanochitosan (NC) film was calculated by quantifying its free radical (DPPH•) scavenging activity during 96 h. Briefly, one piece of NC disc was cut (1 × 1 cm), soaked in 5 mL of DPPH methanol solution (100 µM), and the absorbance (Abs sample) of the solution was analyzed at 30 min, 1 h, 2 h, 4 h, 6 h, 8 h, 24 h, 48 h, 72 h, and 96 h. During the analysis, the solution was kept in the dark (covering with aluminum foil) and the bottles tightly closed to avoid the evaporation of the methanol. Lastly, the absorbance was noted at 517 nm along with a blank sample (Abs Blank) having only DPPH methanol solution. Equation (1) was then applied to estimate the DPPH• scavenging activity of the NC disc:

Inhibition (%) = ((Abs blank − Abs sample)/(Abs blank)) × 100(1)

#### 2.9.2. Quantitative Analysis of Antibacterial Efficacy

Gram-negative bacterium (*Escherichia coli* ATCC 27195) and Gram-positive bacterium (*Staphylococcus aureus* ATCC 25923) were selected to examine the antibacterial activity of the fungal nanochitosan film according to the literature [22]. The NC sample film (1 × 1 cm) was soaked in the test tubes containing the respective Muller–Hinton broth (MHB) media (10 mL) for *E. coli* and *S. aureus* of 1 × 10^6^ CFU/mL, and then placed in a rotary shaker at 37 °C. The optical density at 600 nm (OD 600) of the bacterial culture medium was recorded spectrophotometrically over time at an interval of 2 h up to 6 h to monitor the growth rate of bacteria. The reduction in bacteria was calculated using Equation (2):

Bacterial reduction (%) = 100(B − A)/B(2)

where A was the OD 600 value of bacterial culture medium inoculated with the sample, and B the OD 600 value of bacterial culture medium without the sample.

To increase the interaction between nanochitosan (NC) and the bacteria cell wall, a comparative assay was performed by acidification of NC sample film (1 × 1 cm) dipped 4 h in 96% ethanol solution (pH 4 using HCl solution). Before the antibacterial assay, the acidic film (NC-NH_3_^+^) was previously washed 5 times in absolute ethanol to remove the trace of HCl and dried at 50 °C overnight.

## 3. Results

### 3.1. Characterisation of Fungal Nanochitosan

**Figure 1:**
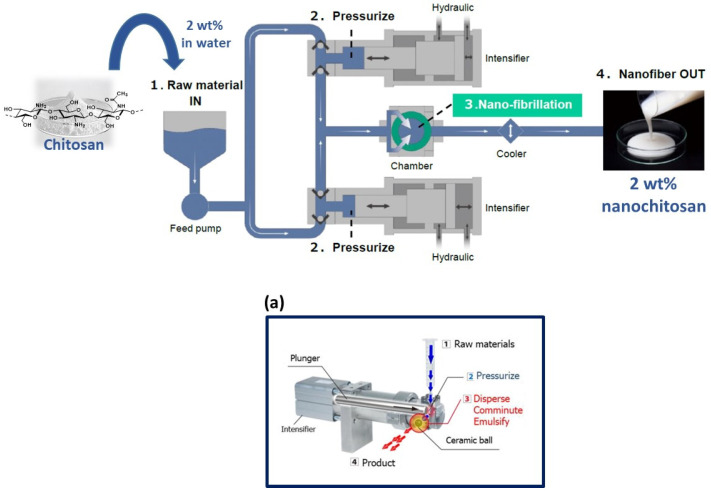
High-pressure water jet system (Star Burst Mini) for the production of the fungal chitosan nanofiber with (**a**) the specific mechanism of Star Burst Mini.

In this present work, we used the high-pressure water jet system technology (Star Burst Mini, Sugino) for the first time on a high molecular weight fungal chitosan (Mw = 400 kDa; Dac = 15.8%) (Figure 1) from *Agaricus bisporus* [6] to generate high-yield (>95%) chitosan nanofibers.

**Figure 2:**
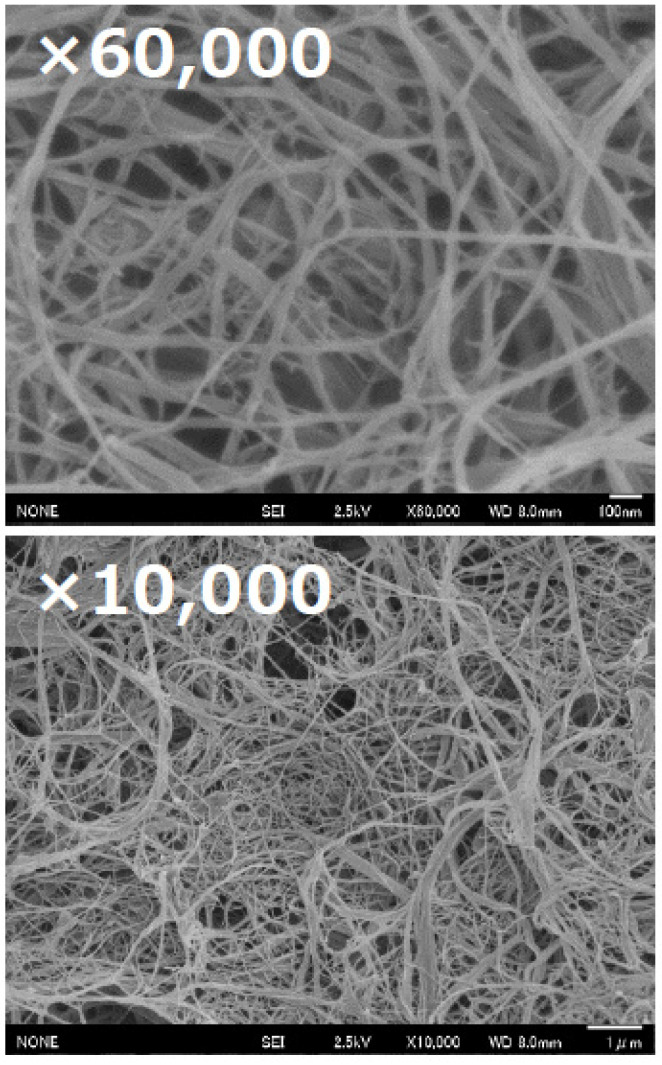
FE-SEM analysis of the fungal chitosan nanofibers (10 passes of treatment) obtained from the high-pressure water jet system (Star Burst Mini).

In this process, we performed 2–20 passes of mechanical treatment with the Star Burst system in water (2 wt% of fungal chitosan concentration) in order to check the nanofibrillation of fungal chitosan. As observed in Figure 2, the nanofiber network was confirmed after 10 passes where the furthermost of the fungal chitosan aggregate forms have been clearly nanofibrillated in nanometer fibers. Not to mention that for the lower number of passes (2 and 5 passes), a low amount of fiber at nanoscale (<100 nm) have been observed and more micron-sized fungal chitosans are present (data not shown).

**Figure 3:**
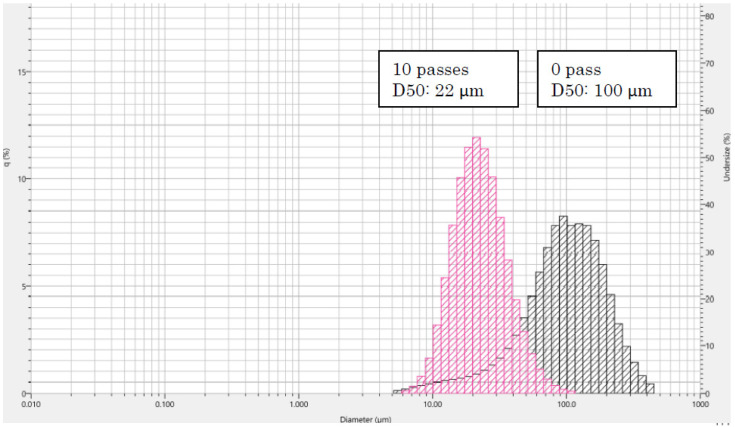
Particle size distribution of the fungal chitosan after 0 and 10 passes of treatment with high-pressure water jet system.

More, additional passes of mechanical treatment (from 10 to 20 passes) did not significantly increase the nanofibrillation where the average nanofibers diameter is in the scale of 20 microns as in the case of 10 passes of treatment where we observed a well-defined particle size distribution of 22 µm (D50) using the Laser Scattering Particle Size Distribution Analyzer (Figure 3).

**Figure 4:**
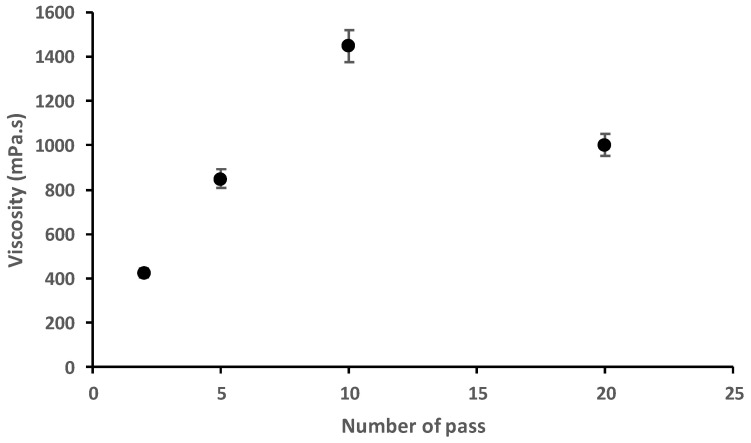
Viscosity of the fungal chitosan nanofiber suspension (1 wt% in water) after 2 to 20 passes of treatment with the high-pressure water jet system.

All these results are consistent with the recent literature indicating that 10 passes of treatment using the high-pressure water jet system seems to be a good technological compromise for the generation of high quantity nanochitosan fibers morphology with high molecular weight and good nanofibrillation of chitosan water suspension as shown by the study of Dutta et al. (2013) and Aklog et al. (2015) describing efficient chitosan nanofiber production using the Star Burst system [20,23]. Moreover, as described in the literature [20,23] the rheological behavior of fungal nanochitosan suspension confirmed the depolymerization of nanochitosan with the high-pressure water jet treatment of more than 10 passes. In fact, in this present work, notable decrease in the viscosity from 1450 mPa.s (pass 10) to 1000 mPa.s (pass 20) for fungal chitosan nanofiber diluted suspension (1 wt% in water) was observed (Figure 4).

This observation is correlated with recent works of Aklog et al. [23] where the molecular weight of crustaceous chitosan was considerably reduced for the number of passes higher than 10. It was particularly shown that the molecular weights of the nanofibers decreased due to the mechanical disintegration of chitosan using a high number of passes. In this study, the Mw decreased from 479 kDa (0 pass) to 63 kDa (50 passes).

**Figure 5:**
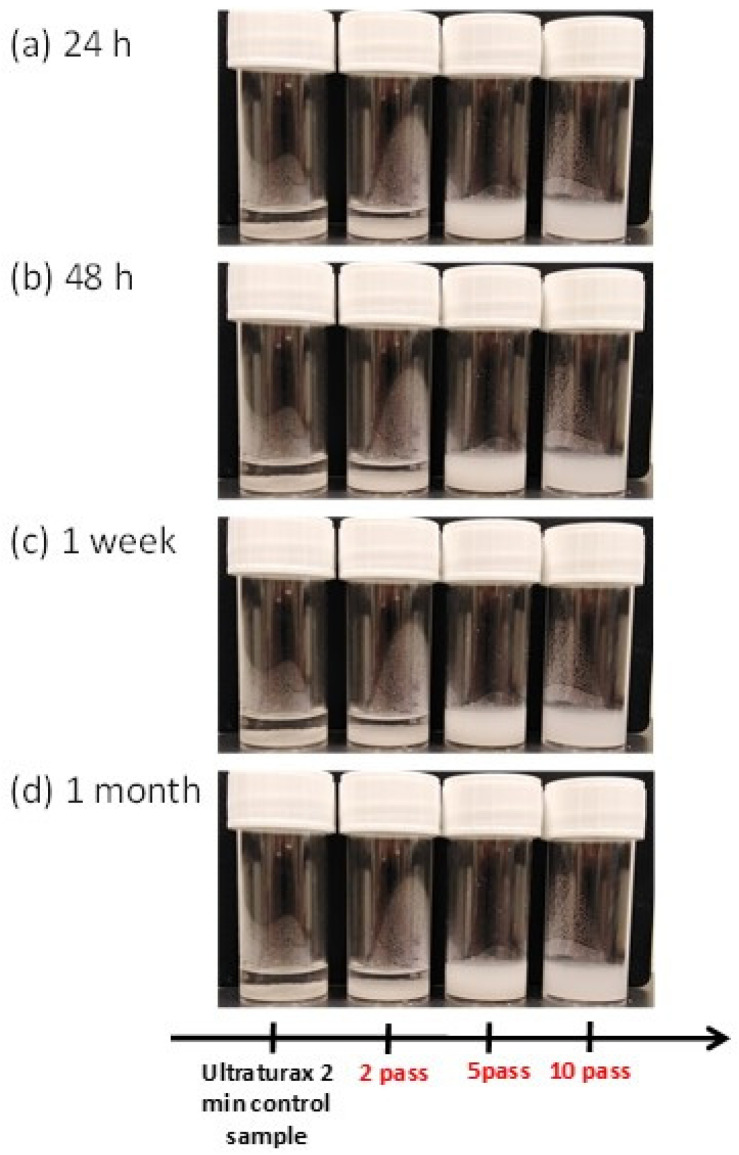
Appearance of fungal chitosan nanofiber suspension (2 wt% in water) during 1-month stockage (4 °C) after 2 to 10 passes of treatment with the high-pressure water jet system. With (**a**) 24 h of storage, (**b**) 48 h of storage, (**c**) one week of storage and (**d**) 1 month of storage.

In our study, the stability of nanochitosan suspension (2 wt% in water) was confirmed after 1 month of storage at 4 °C. Figure 5 shows that no precipitate is formed after one-month storage at 4 °C for 5 and 10 passes of treatment using the high-pressure water jet mechanical process. As well-defined in the literature, chitosan is not soluble and/or fully dispersible in water and needs acid pH (lower than 6) to be dissolved before potential applications [1,2]. In this context, nanofibrillation using the high-pressure water jet system (Star Burst Mini) constitutes a very interesting industrial process to increase the use of fungal chitosan suspension for biomedical and food applications without the use of an acid condition.

**Figure 6:**
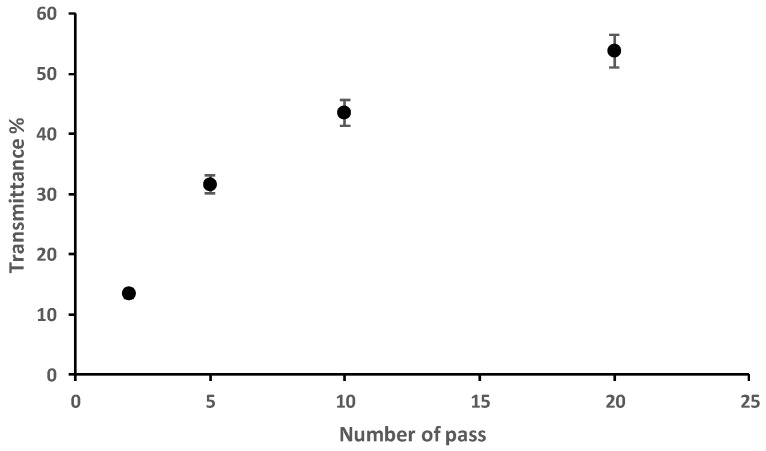
Light transmittance (at 600 nm) of fungal chitosan nanofiber suspension (0.1 wt% in water) after 1 to 20 passes of treatment with the high-pressure water jet system.

In a second step, we analyzed the light transmittances at 600 nm of fungal nanochitosan diluted suspensions at 0.1 wt% in water in order to check the transparency of nanofibers suspension which is known to be strongly correlated with the nanoscale and thickness of fibers dispersion in water [11,12,13,14,15]. As observed in Figure 6, the light transmittance increased with the number of passes to reach around 54% for 10 passes (43.5%) to 53.8% for 20 passes of fibrillation mechanical treatment. These results confirmed the modification of chitosan morphology from micro-sized to nano-sized fibers after using the high-pressure water jet system. Consequently, transparency increased in agreement with our results from FE-SEM analysis and with the main results described in the literature on nanofibers [11,12,13,14,15,21,23].

### 3.2. Characterisation of Fungal Nanochitosan Films

**Figure 7:**
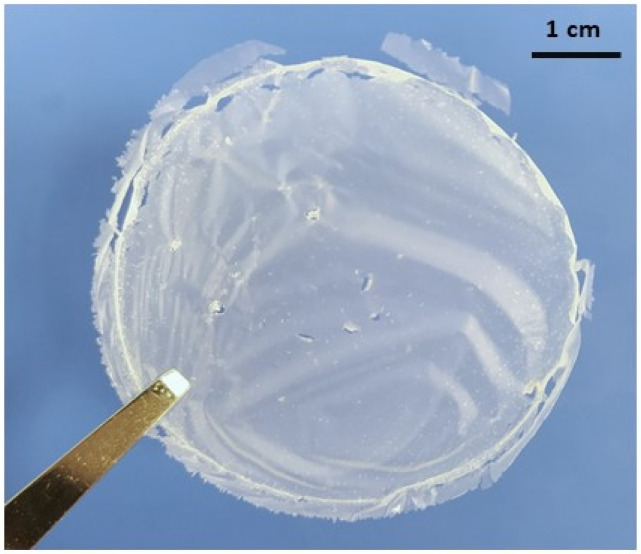
The film from the fungal chitosan nanofiber suspension (0.5 wt% in water) after 10 passes of treatment with the high-pressure water jet system.

It is well known that the main drawback of chitin derivatives such as chitosan is the non-solubility in water leading to low industrial valorization for film or composite material. Generally, to prepare chitosan film, it is necessary to develop film casting strategies for chitosan solution using acid to first dissolve chitosan (at pH lower than 6) before removing the solvent such as acetic acid, lactic acid or HCl [1,2,3,4,5,6,7,8,9,10,11,12,13]. According to literature, the capacity to prepare films using nanofibers suspension such as nanocellulose, nanochitin, and nanochitosan suspension is clearly mentioned since nano-sized fibers are very homogeneously dispersible in water [1,2,11,12,13,14,15,23]. Therefore, we managed the preparation of nanofiber films with a vacuum filtration process of PVDF membrane using fungal chitosan nanofiber suspension (0.5 wt% in water) after 10 passes of mechanical treatment with the high-pressure water jet system (Figure 7). As observed, the fungal nanochitosan film is very homogenous with good transparency and approximate thickness of 50 µm for a mass of around 80 mg which is similar to the literature for the main optimal parameters needed for film applications in food and biomedical fields [13,20,23].

The mechanical properties (E, TS and e%) of the produced nanochitosan film (Figure 7) were investigated. It was then shown that the Young’s modulus (E), the tensile strength (TS), and elongation at break (e%) have been evaluated at 2950 MPa, 50.5 MPa, and 5.5%, respectively. Our mechanical results are in range of similarity to those described by Dutta et al. [20] where for 10 passes of treatment 3535 MPa and 59.9 MPa for E and TS were observed, respectively. According to Aklog et al. [23], the Dac could be a structural key factor influencing the mechanical performance of nanochitosan films. In fact, it was mentioned that for a fully deacetylated high molecular weight crustaceous chitosan, after 10 passes of treatment, the Young’s modulus and the tensile strength were estimated at around 1500 MPa and around 20 MPa, respectively. These lower mechanical properties are probably due to lower molecular weight and shorter nanofibers obtained by the mechanical treatment of a deacetylated chitosan.

### 3.3. In Vivo Biological Activities of Fungal Nanochitosan Films

**Figure 8:**
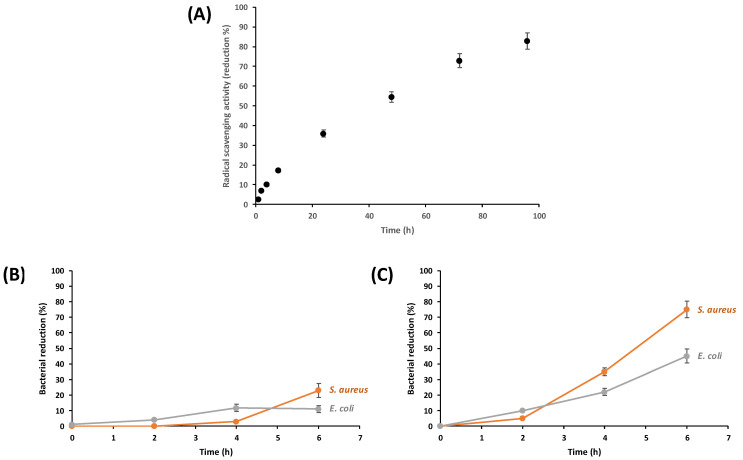
(**A**) Antioxidant properties, (**B**) antimicrobial properties of the film from fungal chitosan nanofiber suspension (0.5 wt% in water) after 10 passes of treatment with the high-pressure water jet system, and (**C**) antimicrobial properties of an acid-activated nanochitosan film (NC-NH_3_^+^).

Biological activities evaluations were performed using the film from fungal chitosan nanofiber suspension (0.5 wt% in water) after 10 passes of treatment with the high-pressure water jet system (Figure 7). In this study, we investigated the main essential biological properties looking for medical applications such as the antioxidant and antimicrobial properties which are very important to prevent bacterial infection [2,7,8,11,13,22,23]. First, as it can be seen in Figure 8A, the radical (DPPH•) scavenging activity of the fungal nanochitosan film increased from 1.21% to 82.84% during 96 h of treatment, indicating then a very good antioxidant effect for long-time contact on nanochitosan fibers with radical molecules model, such as DPPH•.

On the other hand, quantitative analysis of antibacterial efficacy of the same fungal nanochitosan film (Figure 8B,C) was accomplished on Gram-negative bacterium (*Escherichia coli* ATCC 27195) and Gram-positive bacterium (*Staphylococcus aureus* ATCC 25923) which are the main selected bacteria to examine the efficiency of composite film according to the literature [2,6,7,9,11,22]. As largely described in the literature working on antimicrobial effects, it is well known that bacteria absorb and scatter the light, the higher the bacteria concentration, the higher the turbidity [2,6,7,9,11,22]. The absorbance (or optical density) is directly proportional to the bacteria concentration. As shown in Figure 8B, we examined the percentage reduction of *E. coli* and *S. aureus* as a function of time for culture medium inoculated with nanochitosan film for 2 h to 6 h. The nanochitosan (NC) sample film (1 × 1 cm) exhibits 0% bacterial reduction after 2 h incubation for both *E. coli* and *S. aureus*. Bacterial reduction increased to around 11% (±1%) after 4 h and 6h for *E. coli* and, was estimated at 23% after 6 h for *S. aureus* (±1.5%). As mostly discussed in the literature, one of the main mechanisms during antimicrobial activity is without contest the cationic nature of chitosan which interfered efficiently with the negatively charged residues present at the surface of bacteria leading to the cell activity disruption of bacteria and causing sedimentation of bacteria–chitosan aggregate [2,7,8,9,10,24]. Then, to increase the interaction between nanochitosan and the bacteria cell wall, the nanochitosan (NC) sample film (1 × 1 cm) was dipped in 96% ethanol solution at pH 4 using concentrated HCl to increase the presence of cationic function (-NH_3_^+^) at the surface of the nanochitosan film. Subsequently, as observed in Figure 8C, the activated nanochitosan film (NC-NH_3_^+^) possessed higher antimicrobial effect by reducing *E. coli* and *S. aureus* more after 6 h of contact with 45% reduction and 75% reduction, respectively. This effective increasing antimicrobial effect of NC-NH_3_^+^ on Gram(+) and Gram(−) bacteria is extremely interesting for the local prevention of a bacterial infection for medical development applications [2,7,11,22].

## 4. Discussion

From a medical point of view, the synthesis of new active composites such as antioxidant and antibacterial nanomaterials have gained great attention in recent years, exclusively because of the continuous increase in resistance of microbial organisms to the main antibiotics [2,5,7,9,25]. In this context, the production of antibacterial polymer nanocomposites appears essential and crucial for preventing microbial infection for example in biomedical supplies for clinical treatment [25]. Lately, growing interest has been considered for nanomaterial, such as nanofibers (NFs) from abundant biopolymers such as chitin and chitosan, insofar as these polysaccharides are well-defined as sustainable, renewable, biocompatible, biodegradable, and bioactive as antimicrobial agents [1,2,7,9,10]. In the latest nanotechnology advances, the development of functional bioactive chitosan nanomaterial with high and exclusive physical and chemical properties have been provided in the comprehension of antimicrobial compounds for medicinal applications as reported by Jayakumar et al. [11] and correlated by the studies of Rabea et al. [7] on the mode of action and biomedical applications of chitosan as an antimicrobial agent against fungi and bacteria. In this context, we developed a promising industrial low-cost process using a high-pressure water jet system (Star Burst System) allowing one to produce very homogenous nanochitosan fibers (nanoscale size) morphology essential for medical applications [2,7,8,11].

Over the last few years, several publications have shown that chitin and its derivatives chitosan powders could be transformed into nano-sized fibers with different physicochemical and mechanical treatment. Then, lot of methodologies were investigated that generate nanochitosan/nanochitin from crustaceous and cell walls mushrooms with for example the electrospinning technology which allow to produce artificially nanochitosan fibers using chitosan solution (electrically charged fluids) in interaction with an electrically charged metal surface [14,16,26,27]. Nevertheless, this method is not without drawbacks since due to the high polycationic properties of chitosan (as poly-R-NH_3_^+^), it is essential to increase the electrical force and then the final industrial cost for the high-yield production of chitosan nanofibers for biomedical applications [16,28]. Therefore, authors prefer the mechanical methods to disintegrate chitin and chitosan into nano-sized fibers using the nanofibrillation process such as by ultrasonic, grinder technology and atomizing system using effective high-pressure waterjet systems [13,16,17,18,19,20,21,23]. In this industrial consideration, we have investigated the development of a novel efficient antioxidant and antimicrobial nanomaterial based on fungal chitosan through high-pressure water jet system technology (Star Burst Mini, Sugino) applied to the nanofibrillation of fungal chitosan from *Agaricus bisporus*. Chitosans are traditionally obtained by thermochemical treatment of chitin isolated from the crustacean shell wastes. However, this process has some drawbacks such as: (i) environmental issues due to a high amount of pollutant products used and high energy consumption; (ii) limited availability of raw material (crustaceous shells), depending on the seasonal year; (iii) heterogeneity of physicochemical characteristics in the end-chitosan products: degree of deacetylation (DD), degree of polymerization, protein content, among others; and (iv) high production cost [29]. Some traces of allergenic compounds from crustaceans can be present on the final material. Fungal chitosan production is less pollutant than crustacean because it does not contain minerals. The raw material production can be planned throughout the year and, most importantly, the composition of the raw material is reproducible. Thus, the quality and composition of fungal chitosan are more homogeneous than those from crustaceans and also cheaper and present higher potential for pharmaceutical applications.

In our present work, these generated fungal chitosan nanofibers have a very strong potential for the development of nanofibers science and technology for biomedical application because of their excellent micro- and nano-sized structure, good nano-scale morphology, very high surface-to-volume ratio, high water dispersibility, viscosity for long periods of stability (at least one month), and excellent physical properties such as high Young’s modulus (E = 2950 MPa) and high tensile strength (TS = 50.5 MPa) in agreement with all recent works on the nanofibrillation of chitosan using high-pressure waterjet technology [13,20,23].

However, biological activities remain the most significant for their biomedical potential in wound healing application as nanomaterial film with high long-term antioxidant properties and antibacterial effect against *S. aureus* and *E. coli*. In fact, in previous works it was clearly established that during wound healing response, a lot of reactive oxygen species (ROS) played a very crucial role [30]. Accordingly, for wound healing application, antioxidant materials are particularly needed to regulate the main biological balance in ROS regulation [31]. According to Comino-Sanz et al. [30], chitosan is a good candidate for the development of wound healing material due to its highly biocompatible, hemostatic, antibacterial and antioxidant agent. More, chitosan nanofibers have been described as bioadhesive material with good potential for wound dressing applications [32,33]. Consequently, in our study we have clearly established the potential use of fungal nanochitosan films in wound healing treatment by: (i) validating the high long-term (96 h) antioxidant effect of 82.4% which is essential for the regulation of ROS and (ii) obtaining very interesting antimicrobial activity of the nanochitosan (NC) activated fibers (NC-NH_3_^+^) with 75% reduction and 45% reduction observed for *E. coli* (Gram-negative) and *S. aureus* (Gram-positive), respectively, after 6 h of treatment which appears crucial for microbial infection prevention in biomedical applications as largely described in the literature [25].

Interestingly, an important finding from this work is that antibacterial activity can be reached without any acid treatment to dissolve it before the synthesis of nanochitosan films. It is important to notice that the fungal chitosan nanofibers preparation does not include acetic acid use but only water suspension of nanochitosan produced by the high-pressure water jet system, so the antimicrobial activity cannot be due to acetic acid trace but only to the chemical structures of produced nanochitosans (-NH_2_, -NH_3_^+^ and -OH functional groups), as it was well-described in the literature that nanofibers of chitin and chitosan improve their antimicrobial activity in comparison with macrofibers [34,35].

## 5. Conclusions

For biomedical applications, a lot of research has been carried out to generate new bio-sourced and bioactive nanomaterials. In recent years, it was well established that fungal chitosans are undoubtedly a promising natural bioresource for the development of commodity composite products to advance antimicrobial and antioxidant nanomaterial products required for example health care. In this context, nanochitosan materials gained more attention over the last few years by using industrial processes such as a high-pressure water jet system (Star Burst System). Therefore, we believe that these new fungal chitosan nanofibers due to their above-mentioned physicochemical, mechanical, and biological properties such as antimicrobial and antioxidant could be commonly employed as a novel green biomaterial in the immediate future for biomedical applications.

## Data Availability

Not applicable.
